# Violence as a source of pleasure or displeasure is associated with specific functional connectivity with the nucleus accumbens

**DOI:** 10.3389/fnhum.2013.00447

**Published:** 2013-08-13

**Authors:** Eric C. Porges, Jean Decety

**Affiliations:** Department of Psychology, The University of ChicagoChicago, IL, USA

**Keywords:** violence, emotion, pleasure, functional connectivity, nucleus accumbens, insular cortex, orbitalfrontal cortex, reward

## Abstract

The appraisal of violent stimuli is dependent on the social context and the perceiver's individual characteristics. To identify the specific neural circuits involved in the perception of violent videos, forty-nine male participants were scanned with functional MRI while watching video-clips depicting Mixed Martial Arts (MMA) and Capoeira as a baseline. Prior to scanning, a self-report measure of pleasure or displeasure when watching MMA was collected. Watching MMA was associated with activation of the anterior insula (AI), brainstem, ventral tegmental area (VTA), striatum, medial, and lateral prefrontal cortex, orbitofrontal cortex, somatosensory cortex, and supramarginal gyrus. While this pattern of brain activation was not related to participants' reported experience of pleasure or displeasure, pleasurable ratings of MMA predicted increased functional connectivity (FC) seeded in the nucleus accumbens (NAcc) (a structure known to be responsive to anticipating both positive and negative outcomes) with the subgenual anterior cingulate cortex (ACC) and anterior insular cortex (AIC) (regions involved in positive feelings and visceral somatic representations). Displeasure ratings of MMA were related to increased FC with regions of the prefrontal cortex and superior parietal lobule, structures implicated in cognitive control and executive attention. These data suggest that functional connectivity is an effective approach to investigate the relationship between subjective feelings of pleasure and pain of neural structures known to respond to both the anticipation of positive and negative outcomes.

## Introduction

Across animal species, violence is a salient stimulus. It is an important environmental signal with survival consequences, conveying information about potential threats to one's health and physical integrity. Intraspecific violence is a near ubiquitous feature of the environment, and mammals, humans intraspecies, are no exception. Anthropologists agree that violence is pervasive, ancient, infinitely various and a central fact of human life (Whitehead, 2004). Like violence in general, violence in the context of entertainment (e.g., pankration, gladiators, boxing, etc.), is reported throughout the historical record of human behavior as one of the oldest forms of entertainment (Schechter, 2005). Examples of violent entertainment exist throughout contemporary society and are widely sought out; indicating that within the context of entertainment, a sizable portion of the population is attracted to viewing violence.

During the past decade, a number of functional neuroimaging studies have investigated the neural response while observing other individuals being hurt (Morrison et al., 2004; Jackson et al., 2005a; Cheng et al., 2007; Gu and Han, 2007; Lamm et al., 2007a,b; Moriguchi et al., 2007; Zaki et al., 2007; Michalska et al., 2013), and devoid of social context these studies have consistently revealed the activation of a specific neural network. This work implicates several brain regions that overlap with regions involved in processing physical pain, including the anterior cingulate cortex (ACC), anterior midcingulate cortex (aMCC), anterior insula (AI), and periaqueductal gray (PAG). Some of these studies have also reported involvement of the somatosensory cortex (Cheng et al., 2007, 2008; Moriguchi et al., 2007; Benuzzi et al., 2008; Lamm and Decety, 2008; Wood et al., 2010). Together, these regions are thought to be responsive to aversive stimuli, directing attention toward and supporting a representation of these stimuli, as well as supporting a response to a potential threat in the environment (Ogino et al., 2007; Benuzzi et al., 2008; Akitsuki and Decety, 2009; Decety and Michalska, 2010). Although this ensemble of regions recruited when one watches or experiences pain (sometimes referred to as the pain matrix) is well established (for review see Lamm et al., 2011), much less is known for social situations that are both violent and socially appropriate.

A limited number of studies have investigated neural responses to violence in the context of socially acceptable entertainment such as television shows (e.g., Murray et al., 2006a). In these studies, a similar pattern of neural recruitment was observed, as in previous research with non-entertainment stimuli depicting pain and injury, with the notable inclusion of the striatum. In rodents the nucleus accumbens (NAcc), a structure within the striatum, has populations of cells that are selectively responsive to the anticipation of aversive outcomes (Badrinarayan et al., 2012). Other investigations have identified distinct regions within the NAcc to be particularly responsive to uncertain, but salient events, with outcomes that could be either positive or negative (Anselme et al., 2013). In humans, while no functional segregation has been demonstrated, the NAcc has similarly been reported to be particularly responsive to the anticipation of salient events where the outcome is uncertain but could be either positive or negative (Jensen et al., 2003; Cooper and Knutson, 2008).

NAcc recruitment associated with both positive and negative outcomes is not unexpected, as it is still unclear whether separate neural systems are involved in aversive and appetitive processing in the brain. In fact, at the systems level, there is extensive overlap throughout the neural circuitry and chemistry involved in processing appetitive and aversive stimuli (Leknes and Tracey, 2008). Recently, progress has been made in disentangling the specific circuits that underlie this complex behavior in animal models (Smith et al., 2011).

Describing a psychological construct as synonymous with neuroanatomy, such as “pleasure” always being concurrent with NAcc recruitment, obscures the nuances of brain reactivity. For example, when rodents were placed in a stressful or threatening environment, the regions of the NAcc that were activated in response to aversive stimuli, roughly double in size compared to baseline (Reynolds and Berridge, 2008). However when rodents were housed in their preferred, safe environment, regions associated with appetitive behaviors expanded dramatically while those associated with responses to fear shrunk to one third of their baseline size.

Given the importance of the information conveyed in observed violence, neural structures sensitive to salience, such as portions of the ventral striatum including the NAcc, should be responsive to viewing violence independent of the subjective appraisal. Only after this appraisal, which occurs in the context of the viewer's relationship with the stimuli, can an evaluation and behavioral strategy be generated to observed violence. These behavioral responses, emerging from the connectivity of neural structures' responsive to salience, would be dependent on the individual's bias to perceive violence as pleasurable or not pleasurable.

Previous functional neuroimaging research has shown that a small shift in context or in the instructions given to a subject modulates neural responses to the observation of another in pain; these shifts have been observed both centrally and peripherally. For example, inducing an individual to adopt the perspective of another person experiencing pain increases the magnitude of facial muscle contractions associated with first person pain, and was associated with increases heart rate (Lamm et al., 2008). Presenting a participant with stimuli depicting a person being injured by another under two instructions sets—“imagine you are harming the other” and “imagine you are being harmed by the other”—produces a relatively larger response in pain matrix, as well as in regions associated with autonomic arousal (Decety and Porges, 2011). More importantly, in the previous study, the most reliable and significant changes in brain response were detected in functional connectivity (FC) analyses.

The work cited above has either implicitly or explicitly characterized observed violence as strictly aversive stimuli (Jackson et al., 2005a, 2006; Lamm et al., 2007a; Lamm and Decety, 2008) and demonstrated changes in functional recruitment based on shifting the context of the violence within subject (Lamm et al., 2008; Decety and Porges, 2011) or selecting subject populations that would contextualize the violent stimuli in different ways, such as physicians viewing a medical procedure as compared to lay subjects viewing the same procedure (Cheng et al., 2007). However, violence has a long history of being a source of entertainment and pleasure, and today there are still numerous socially appropriate ways for people to watch and consume violence, indicating that observing violence is not always aversive, but can also be desired and contain pleasurable aspects for the viewer. These appetitive aspects are apparent in nearly all forms of popular media: television, movies, video games, etc. where violent content is overtly marketed. We hypothesize that the neural mechanisms engaged when an individual views violence in the context of entertainment they derive pleasure from viewing will be functionally distinct from those activated when an individual observes they find unpleasurable to view.

The present study tests this hypothesis by focusing on the dynamic response of the NAcc and the functional relationship this region has with other neural targets, as a function of individual differences in the relationship to a given stimulus. To test this hypothesis, the study displayed violent stimuli to participants, who had previously reported the degree of pleasure or displeasure they derived from watching similar videos. Based on this self-reported measure, a FC analysis was run, seeded from the NAcc with pleasure ratings used as a predictor. Drawing on previous research, we anticipated that whole brain responses would include the activation of regions implicated in the perception of pain in others (Jackson et al., 2005b; Murray et al., 2006a; Decety and Porges, 2011). We further predicted that findings in the FC analysis seeded from the NAcc would increase with regions associated with positive somatic and visceral representations and pleasurable states, including the AI (Damasio and Damásio, 1994; Craig, 2009) and frontal cortex (e.g., Moll et al., 2007).

## Methods

### Participants

Forty-nine male individuals were recruited from the Chicago metropolitan area (mean age = 25 years; range = 18–35 years). Flyers and online advertisements were posted in the region that targeted individuals who either did or did not enjoy watching Mixed Martial Arts (MMA). Participants had no neurological diagnosis and had normal or corrected to normal vision. Participants' written informed consent was obtained, and this study was approved by the Institutional Review Board at the University of Chicago.

### Materials

Stimuli consisted of 10 s video clips of the target condition (MMA) or control (Capoeira), 20 clips per condition. MMA is a full contact combat sport similar to boxing and kick boxing, but also involves the use of other martial arts techniques such as wrestling. MMA contestants utilize these tools to knock unconscious, force their opponent to “give up,” or be designated the winner by a panel of judges. MMA has grown increasingly popular since its start in 1993, with the parent company of UFC valued at approximately $1 billion in 2008 (Gregory and Osborne, 2009). MMA is a sport regularly shown on both general broadcast television and basic cable and all clips used in this study were in line with standards for these media. Non-MMA, control condition stimuli consisted of Capoeira, a form of dance, derived from Brazilian martial arts, which like MMA involves kicks and hand and arm strikes, such as punches, between two participants of the same gender, but no contact is made between participants and no injury or intent to injure takes place. This control condition was selected because it was non-violent, but in all other ways, is very similar in physical and visual characteristics to MMA.

### Procedure

To ensure all participants were run under consistent conditions, the same 2 investigators, dressed in white lab coats, were responsible for all subject interactions. Prior to scanning, subjects were asked to report “How pleasurable do you find watching MMA?” on a 5-point Likert scale ranging from −2 “extremely unpleasurable” to 0 “Neither pleasurable or unpleasurable” to + 2 “Most pleasurable” (*m* = 0.45, std = 1.04). Stimuli were presented using E-Prime 2.0 Professional (Psychology Software Tools, Inc., Pittsburgh, PA) and displayed in-scanner via an Avotec rear projection system (Avotec Inc., Stuart, FL). Prior to functional runs, subjects were shown sample stimuli of both classes in the manner the actual task would employ. During functional runs, each participant viewed one of four possible stimuli permutations. Two of the permutations were randomly generated with manual oversight to prevent the same class of stimuli being displayed more than twice in a row; these permutations were inverted for a total of four permutations. Prior to the onset of all stimuli, a one-word text cue describing the stimuli that were to appear was displayed for 1 s. A 10 s inter-block interval preceded and followed all video clips. Due to the engaging nature of the task and the anticipation of motor, motor planning, and somatosensory recruitment, a decision was made to give participants no task during functional runs other than to attend to the stimuli. Compliance was confirmed via in scanner camera that allowed for observation of subjects eyes. Following collection of functional data, a structural scan was collected.

### MRI scanning parameters and analysis

#### Functional acquisition

Whole brain functional MRI data were collected with an 8-channel Phillips Sense head-coil in a Philips Achieva 3T scanner using Phillips provided T2^*^ weighted EPI sequence (32 slices, TR 2 s, TE 25 ms, FOV 240 × 240 × 127.5 mm, 80 × 80 × 32 mm matrix, flip angle 80°, in plane resolution of 3 × 3 mm, slice thickness 3.5 mm, 0.5 mm skip). To recover signal in the orbital frontal region, a Philips provided Z-Shim sequence was utilized.

#### Structural MRI

Anatomical scans were acquired in the axial plane using a Philips SENSE-Ref, T-1 weighted sequence (300 slices, 1.2 mm, −0.6 mm gap, FOV: 250 × 250 × 180 mm, 240 × 240 × 300 matrix).

#### Image processing and analysis

Using SPM8 (Wellcome Department of Imaging Neuroscience, London, UK) in Matlab (Mathworks Inc., Sherborn, MA, USA), functional volumes were motion realigned, with movement parameters saved for use at the first level as repressors of no interest. Functional images were co-registered to the segmented structural images (gray matter, white matter, and cerebral spinal fluid), and these structural images were normalized to the MNI template with these normalization parameters applied to the co-registered functional images. Normalized functional images were resliced as 2 mm isotropic voxels and smoothed with an 8 mm full-width at half-maximum Gaussian kernel. Hemodynamic responses were assessed by setting up fixed effects general linear models (GLM) for each subject. Regressors of interest modeling the experimental conditions and the cue displayed prior to all stimuli, epochs were defined and convolved with the canonical hemodynamic response function (HRF). All models included a high-pass filter with a cut-off at 128 s in order to remove scanner drifts. Following model estimation, contrasts were calculated for each subject to assess differences between conditions, and, in relation to the implicitly modeled fixation, baseline data were assessed. The resulting first-level contrast images were entered into second-level random effects (RFX) analyses to assess differences between conditions with population inference. Activation differences between conditions were assessed using a voxel-level threshold of *p* = 0.01 and a spatial extent threshold of *k* = 10, corrected for multiple comparisons across the whole volume using the false discovery rate (FDR, *p* < 0.05) approach (Genovese et al., 2002). Results were visualized in xjView toolbox (http://www.alivelearn.net/xjview) and MRIcron (http://www.mccauslandcenter.sc.edu/mricro/mricron/install.html).

ROI analysis of activation while watching MMA vs. an implicitly modeled baseline condition (fixation cross) was extracted. This was conducted using RFXplot (Gläscher, 2009), with a bilateral, 10 mm sphere mask created in WFU Pickatlas (Maldjian et al., 2003) of the NAcc. Results were extracted in percent signal change. This sphere was bilateral as laterality of activation has not been widely reported in previous investigations of NAcc activation to either reward or salience (Cooper and Knutson, 2008). The mask was centered at widely reported MNI coordinates for the NAcc ±10, 12, −2 (Luijten et al., 2012).

FC analysis was performed using the Conn FC toolbox (Whitfield-Gabrieli and Nieto-Castanon, 2012), using images preprocessed as previously described in SPM8. The Conn toolbox employs “aCompCor,” anatomically informed component-based noise correction, to correct for physiological and other sources of noise by regressing signal from the white matter and cerebral spinal fluid as well as movement parameters (Behzadi et al., 2007). FC was assessed using how pleasurable subjects found watching MMA as a covariate for a “seed to voxel” methodology; this produced Fisher transformed correlation coefficients for all voxels in the whole brain relative to same bilateral 10 mm sphere seed region used for NAcc region in the ventral striatum in the ROI analysis. A whole brain Family Wise Error (FWE) corrected threshold of *p* < 0.05 was used to identify significant clusters.

## Results

### Whole brain analysis

#### MMA>control

**Figure 1:**
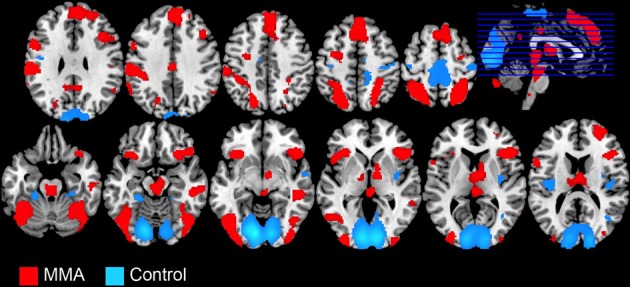
**Whole brain analysis showing BOLD signal changes in 49 male participants while watching videos depicting a full contact, Mixed Martial Arts (MMA, in red) vs. control videos depicting Capoeira (Control, in blue)**.

The whole-brain analysis, FDR corrected at *p* < 0.05, contrasting the MMA condition vs. the control condition (Capoeira) revealed greater activation in a large number of clusters extending from the supplementary motor area (SMA) to the medial aspect of the superior frontal gyrus (SFG), to right dorsolateral prefrontal cortex (dlPFC). A large activation was detected in the right middle temporal gyrus extending to the superior temporal sulcus. Bilateral activation of the AI, precentral, post central gyrus, and superior parietal and temporal parietal junction (TPJ) were observed as well as bilateral regions in the visual cortex and cerebellum. Posterior and middle cingulate cortex were robustly recruited as well as extensive thalamic and brainstem recruitment such as the PAG, ventral tegmental area (VTA), and regions in the striatum including the head of caudate (see Table 1 and Figure 1).

**Table 1:** **Brain regions that show a significant hemodynamic increase when participants watched MMA videos as compared with control videos (Capoeira)**.

**Region**	**Abbreviation**	**Side**	***x***	***y***	***z***	***t*-value**
Anterior insula cortex	AIC	l	−36	24	−8	4.82
Anterior insula cortex	AIC	r	46	22	6	5.41
Anterior thalamus		r	10	−4	−2	5.09
Brainstem		l/r	−6	−20	−20	4.76
Brainstem		r	8	−14	−12	3.65
Brainstem		l/r	−2	−22	−20	4.74
Caudate head	Ventral striatum	r	8	10	4	3.84
Caudate head	Ventral striatum	r	10	8	2	3.83
Caudate body		l	−8	2	12	2.81
Dorsal lateral prefrontal cortex	DLPFC	r	28	50	18	4.56
midcingulate cortex	MCC	l/r	−4	−22	36	4.04
Inferior frontal gyrus	IFG	r	38	24	−20	4.49
Inferior parietal lobule		l	−38	−40	46	3.71
Inferior parietal lobule		r	30	−44	56	5.25
Medial frontal gyrus	MFG	r	54	16	30	3.15
Medial frontal gyrus	MFG	r	38	20	32	4.63
Midtemporal gyrus	STS	r	52	−32	−10	5.11
Periaqueductal gray		l/r	2	−28	−8	3.41
Posterior cingulate cortex	PCC	l/r	4	−50	30	3.56
Postcentral gyrus		r	52	−22	34	3.1
Postcentral gyrus		l	−56	−24	32	7.31
Precentral gyrus		l	−42	−2	46	2.89
Precentral gyrus		r	32	−8	58	3.51
Superior frontal gyrus	SFG	l/r	4	38	50	4.54
Superior medial frontal cortex	SupMPFC	l/r	2	46	40	5.11
Superior medial frontal cortex	SupMPFC	l/r	4	52	24	4.88
Supplementary motor area	SMA	l/r	8	24	56	4.39
Superior parietal lobule	SPL	l	−28	−58	62	8.3
Superior parietal lobule	SPL	r	26	−60	60	7.99
Temporal parietal junction	Supramarginal gyrus	l	−62	−48	36	3.89
Temporal parietal junction	Supramarginal gyrus	r	58	−44	34	2.86
Temporal parietal junction	Supramarginal gyrus	r	54	−56	34	3.46
Temporal pole		r	44	6	−42	3.24
Ventral tegmental area	VTA	l/r	−2	−22	−20	4.74

L, left hemisphere; R, right hemisphere. MNI (x, y, z) coordinates.

#### Control>MMA

A whole brain analysis of the control condition (Capoeira) greater than MMA, FDR corrected at *p* < 0.05, revealed bilateral posterior insula cortex, precentral gyrus, and parahippocampal gyrus, were more active. In addition, increased activation was seen in regions of the visual cortex and a large cluster focused in the posterior portions of the SMA that extended to the bilateral postcentral gyrus, extending on the right side to the superior temporal gyrus.

### Self reported pleasure from MMA

Subjects' reports of the pleasure they derived from watching MMA was used as a covariate of interest in our in the whole brain analysis, FDR corrected at *p* < 0.05, *k* = 5. No regions were significant.

### ROI analysis

**Figure 2:**
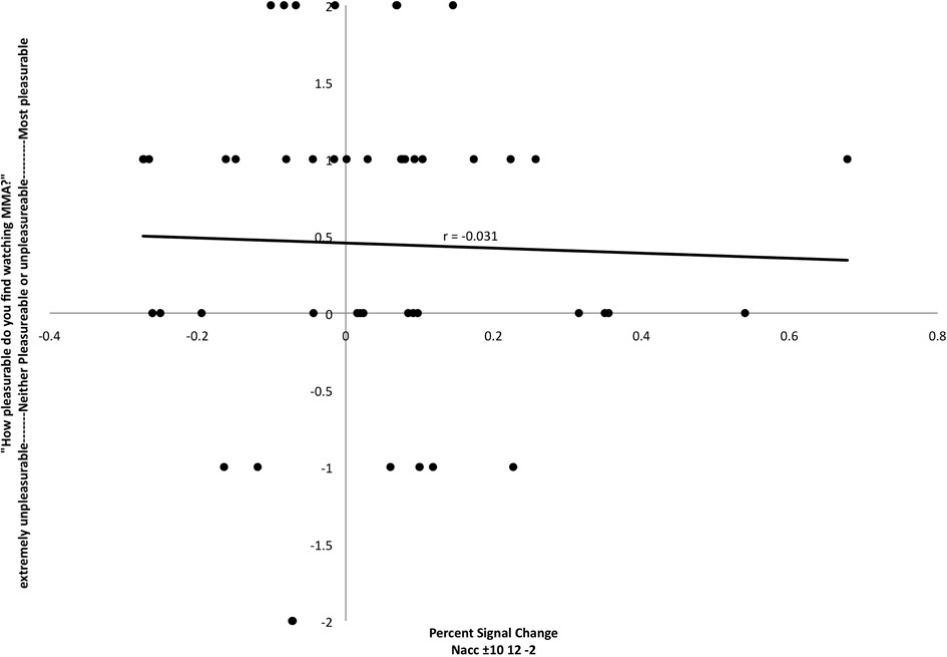
**Relationship between participant's reports of pleasure from watching MMA and percent signal change in the nucleus accumbens (MNI coordinates: ±10, 12, −2)**. Each point corresponds to a single subject's percent signal change on the *X* axis and self-reported pleasure or displeasure rating on the *Y* axis.

In preparation for the FC analysis, a region of interest analysis was extracted for bilateral NAcc against baseline. Activation in that region was not correlated with self-reported pleasure from watching MMA (*r* = −0.031) (Figure 2).

### Functional connectivity

**Figure 3:**
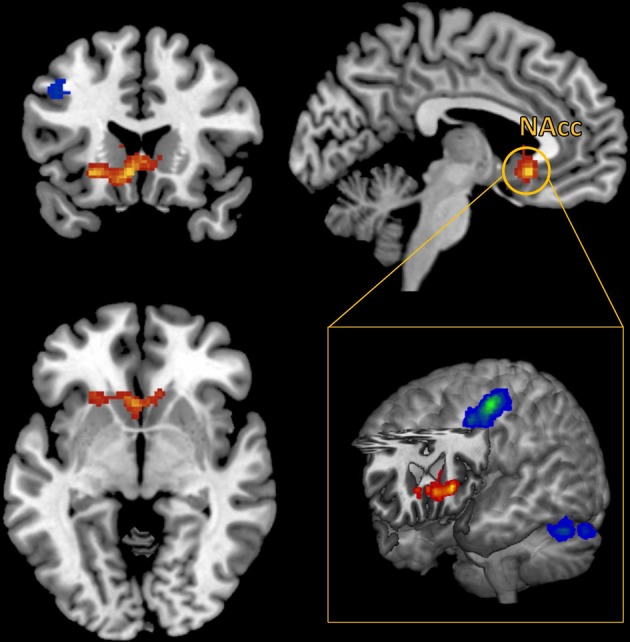
**Functional connectivity seeded in the nucleus accumbens (MNI coordinates: ±10, 12, −2) associated with pleasurable ratings (in red) and connectivity associated with unpleasurable ratings (in blue)**.

A whole brain, FWE corrected threshold of *p* < 0.05 was used to identify significant clusters. A large cluster that extended from the subgenual cingulate cortex to the left anterior insular cortex (AIC) increased in connectivity with the NAcc as self-reported pleasure from watching MMA increased (Table 1). Conversely, as self-reported pleasure from watching MMA decreased, multiple clusters emerged identifying a cluster extending over the left dlPFC, another in the right superior parietal lobule, and a third in the left hemisphere of the cerebellum (Table 2; Figure 3).

**Table 2:** **Regions that show greater effective functional connectivity seeded in NAcc**.

**Brain region**	**MNI (*x, y, z*)**			***k***	***t*-value**
**GREATER FUNCTIONAL CONNECTIVITY WITH NAcc, PLEASURE**					
Left anterior insula/subgenual cingulate cortex	−24	24	−6	363	3.94
**GREATER FUNCTIONAL CONNECTIVITY WITH NAcc, DISPLEASURE**					
Left dorsolateral prefrontal cortex	−48	12	44	303	6.67
Right superior parietal	30	−72	46	185	4.2
Left cerebellum	−36	−58	−26	243	4.96

## Discussion

This study was designed to investigate how the neural response and shift in FC seeded from the NAcc are modulated by the relative pleasure individuals experience when watching violent videos. The violent videos selected were MMA, chosen based of their intensity, pervasiveness, and popularity, as well as the wide range of variability regarding how pleasurable subjects perceived the stimuli.

The whole brain analysis level, contrasting MMA vs. the control condition, identified a pattern of brain response consistent with a large literature that has previously documented the neural response to observing or imagining pain and injury in others. Regions recruited included the midcingulate cortex, SMA, PAG, AI, and somatosensory cortex (Akitsuki and Decety, 2009; Decety et al., 2009; Decety and Michalska, 2010; Decety and Porges, 2011; Lamm et al., 2011). In addition, we observed the involvement of regions implicated in theory of mind, goal oriented attention, and executive function (Corbetta et al., 2008), including a parietal cluster that encompassed the TPJ (Decety and Lamm, 2007) and a cluster over the dorsolateral cortex (Meyer et al., 2012). Regions implicated in autonomic arousal, such as the brainstem and midcingulate cortex (Critchley, 2009), were also activated. Finally, the orbitofrontal cortex, which plays a critical role in emotion and decision-making, but is also responsive to both positive and aversive stimuli (Rolls et al., 2003), and the temporal pole, a region that binds visceral emotional information to perceptual information (Olson et al., 2007), were also detected at the whole brain level, these areas have previously been reported to be responsive to strongly valenced stimuli, such as unpleasant images (Aldhafeeri et al., 2012) from the International Affective Picture System (Lang et al., 1997). Compared to previous neuroimaging research that employed violent, though social acceptable, entertainment with non-violent entertainment (e.g., Murray et al., 2006a), our study found many of the same regions of increased hemodynamic activity including in visual areas, thalamus, caudate, and precentral gyrus. One notable difference, however, is the lack of amygdala activation in response to MMA. While this structure that plays a primary role in the detection and appraisal of biologically relevant stimuli (not restricted to fear) has been widely reported as being responsive to the observation of violence (e.g., Mathiak and Weber, 2006; Decety et al., 2012), or even imaging harming another person (Decety and Porges, 2011), it is important to note that our study used very well matched control stimuli (Capoeira) that contained a large amount of extremely intense and similar visual information, with the main difference from MMA being the lack injury or intent to injure. This is in contrast to the previous literature (e.g., Murray et al., 2006a) which utilized control conditions with dramatically less visual activity, such a National Geographic program made for a child audience. In our study, both MMA and Capoeira, evaluated relative to a fixation cross, produced robust activation in the amygdala.

The stimuli used in the Capoeira control condition were, as in the MMA condition, extracted from commercial videos. Unavoidably, this resulted in some variability between the stimulus classes, with Capoeira stimuli containing a slightly more variable range of environments than MMA, which was always conducted in an environment with consistent features (an octagon shaped cage). In addition, the MMA stimuli tended to have a tighter focus on the participants and slightly fewer kicks and punches than the Capoeira stimuli. These differences, may account for the regions that were more active during the control condition relative to MMA. For example, the posterior region of the parahippocampal gyrus, that we report being more active in this condition than in MMA, has been previously reported to be preferentially responsive to novel visual scenes (Epstein et al., 1999; Epstein, 2011), of which the Capoeira stimuli contained more than the MMA stimuli. Additionally, greater activations during the Capoeira stimuli were observed in the precentral gyrus, a region that, while recruited during motor generation, is also sensitive to the observation of human movements (Servos, 2002; Grosbras et al., 2012). Finally the posterior insula also showed greater activation to the control condition. Though the activation of this region has been widely reported to be involved in nociception, but not the observation of pain in others (Lamm et al., 2011; Garcia-Larrea, 2012), in this study is best accounted for by its role supporting eye movements in complex visual scenes (Blurton et al., 2012; Indovina et al., 2013), a possible consequence of the increased visual motion in the control condition. When activity at the whole brain analysis was covaried with subjects' reported pleasure when watching MMA (data corrected for multiple comparisons), no cluster survived.

Given our predictions regarding the role of the NAcc in response to the perception of highly salient stimuli that have survival implications, an ROI analysis extracted from a bilateral NAcc mask for the MMA condition was performed. The extracted values were then covaried with subjects reported pleasure values from watching MMA. At this level of inquiry, no relationship between the HRF in the NAcc and reported pleasure scores was found (Figure 2). This finding mirrors published studies reporting that this region has a comparable response to the anticipation of both positive and negative outcomes (Cooper and Knutson, 2008; Carter et al., 2009). There is some electrophysiological evidence in animal models that there is a degree of functional segregation for appetitive and aversive responses in the NAcc (Reynolds and Berridge, 2002; Anselme et al., 2013), but perhaps due to limits of spatial resolution in fMRI, this has not yet been demonstrated in humans.

While pleasure ratings were not correlated with brain activity at the whole brain level, they did predict FC seeded in the NAccs. More interestingly, using reported pleasure from MMA as a covariate in the FC analysis revealed a unique pattern of brain activation. Pleasure, while watching MMA, predicted increased connectivity in the subgenual region of the ACC as well as the left AI. The subgenual region has extensive dopaminergic projections from the mesolimbic pathway (Gaspar et al., 1989) and has been previously reported to be responsive to viewing one's offspring (Bartels and Zeki, 2004), making charitable donations (Moll et al., 2006), and has been shown to reflect guilt (Zahn et al., 2009; Green et al., 2012). More generally, it has been associated in animal models with social attachment and pair bonding (Insel and Young, 2001). In patients with major depressive disorder, hypoactivity has been reported in this region (Drevets et al., 1997), and remission of symptoms have been associated with a normalizing of this region's function (Ressler and Mayberg, 2007). The increased FC between the NAcc and this subgenual prefrontal cortex, predicted by self-reported pleasure when watching MMA, may be evidence of other oriented pleasure and perhaps simultaneously guilt around enjoyment derived from the pain of another.

The cluster in the subgenual cingulate cortex extended to and had a second distinct peak in the left AIC. Recent work has begun to integrate the wide range of conditions under which activation of the AIC has been reported in functional neuroimaging studies. This work suggests that the AIC is central to understanding and representing one's own physical and somatic state (Craig, 2009). Furthermore, in our study, the connectivity with the AIC was found only in the left hemisphere, which has been associated with positive affect, appetitive behavior, and group affiliated emotion (Craig, 2005).

Conversely, displeasure while watching MMA predicted increased connectivity in the left dorsolateral cortex and the right superior parietal lobule. Interestingly, these two regions are recruited when physicians watch a painful medical procedure taking place, and this has been interpreted as a sign of top-down regulation and modulation of the aversive aspects of the procedure (Cheng et al., 2007). In addition, supporting the interpretation that recruitment of these regions supports executive function, these regions belong the dorsal frontoparietal, goal directed attention network (Corbetta and Shulman, 2002; Corbetta et al., 2008). It seems that participants, who dislike watching MMA either engage in top-down emotion regulation or, at the least, experience a more effortful appraisal of the stimuli.

In conclusion, when individuals watch violent videos, the overall pattern of brain response is not the most informative source of information in predicting the extent to which one derives pleasure or displeasure. Rather, the connectivity seeded in the NAcc, a structure known to be responsive to salient information in the environment (Smith et al., 2011), demonstrates clear and distinct responses depending on the relationship of the perceiver to the stimuli.

## Future directions

Since an objective of this study was to use ecologically valid stimuli, it was necessary to use videos extracted from commercial broadcast in conjunction with longer stimuli durations. In the future, an event-related design, where temporal dynamics of the response could be better established would be even more informative, and provide additional information. For example, based on work that has attempted to decouple the responses of the ventral striatum to both the anticipation of positive and negative outcomes as well as the outcome itself (Cooper and Knutson, 2008), one would predict that pleasure would have little impact on the response to the anticipation of violence, but it would have significant impact on during sustained observation of violence. Our study utilized stimuli containing a dyad involved in a violent interaction and the influence of these stimuli on the FC of anatomy in a dopaminergic pathway. The response of this neuroanatomy to social conflict, has previously been demonstrated to be influenced by a participants' implicit need for power (Schultheiss and Schiepe-Tiska, 2013). The inclusion of a measure of need for power would likely have accounted for some of the variability in the responses to violent stimuli. Furthermore, the explicit measure of participant's pleasure derived from watching MMA used in the current study could be complemented by an implicit measure of need for power. Finally, our study intentionally restricted the sample to male participants within a narrow age range and geographic region, future studies would benefit from including female subjects and participants from different cultures representing a broader age range.

### Conflict of interest statement

The authors declare that the research was conducted in the absence of any commercial or financial relationships that could be construed as a potential conflict of interest.
